# Senescence in Wound Repair: Emerging Strategies to Target Chronic Healing Wounds

**DOI:** 10.3389/fcell.2020.00773

**Published:** 2020-08-11

**Authors:** Holly N. Wilkinson, Matthew J. Hardman

**Affiliations:** Centre for Atherothrombosis and Metabolic Disease, Hull York Medical School, University of Hull, Hull, United Kingdom

**Keywords:** senescence, ageing, diabetes, wound healing, senolytics

## Abstract

Cellular senescence is a fundamental stress response that restrains tumour formation. Yet, senescence cells are also present in non-cancerous states, accumulating exponentially with chronological age and contributing to age- and diabetes-related cellular dysfunction. The identification of hypersecretory and phagocytic behaviours in cells that were once believed to be non-functional has led to a recent explosion of senescence research. Here we discuss the profound, and often opposing, roles identified for short-lived vs. chronic tissue senescence. Transiently induced senescence is required for development, regeneration and acute wound repair, while chronic senescence is widely implicated in tissue pathology. We recently demonstrated that sustained senescence contributes to impaired diabetic healing via the CXCR2 receptor, which when blocked promotes repair. Further studies have highlighted the beneficial effects of targeting a range of senescence-linked processes to fight disease. Collectively, these findings hold promise for developing clinically viable strategies to tackle senescence in chronic wounds and other cutaneous pathologies.

## Introduction

Senescence, a seminal discovery of Hayflick and Moorhead (1961), is a defined process that globally regulates cell fate. Cellular senescence is traditionally described as a terminal stress response, whereby cells are triggered to undergo stable and essentially irreversible cell cycle arrest following initiation by a diverse range of stress-inducing stimuli (Hernandez-Segura et al., 2018). Indeed, this process acts as an autonomous anti-tumour mechanism, halting incipient neoplastic transformation (Faget et al., 2019). Yet, senescent cells can be found in non-cancerous tissues, accumulating exponentially with increasing chronological age (Hudgins et al., 2018; McHugh and Gil, 2018). These non-proliferative cells retain metabolic capabilities, exhibiting a hypersecretory phenotype (Coppé et al., 2010). It has recently been shown that some senescent cells may even engulf their neighbouring cells, for a survival advantage (Tonnessen-Murray et al., 2019). These profound functional behaviours, identified in cells long thought to be non-functional, pose new questions around their tissue roles and consequences. This review will explore emerging roles for cellular senescence in normal and pathological wound repair, highlighting areas of potential therapeutic opportunity.

## Senescence as an Anti-Proliferation Mechanism

It was originally thought that only mitotic cells, which may be highly proliferative, or spend large periods of time in quiescence, undergo senescence (Campisi and di Fagagna, 2007). This view has since been challenged, as features of senescence are observed in some differentiated cells (Jurk et al., 2012; von Zglinicki et al., 2020). The major age- and stress-related processes that induce cellular senescence include replicative exhaustion (Hayflick and Moorhead, 1961), mitogenic signals (Tchkonia et al., 2013), oxidative stress (Passos et al., 2010), DNA breaks (Di Micco et al., 2006), and epigenomic damage (Pazolli et al., 2012). These stressors subsequently initiate anti-tumourigenic networks, controlled by transcriptional regulators such as p53 (Vousden and Prives, 2009). p53 directly transactivates the cyclin dependent kinase (CDK) inhibitor, p21, to inhibit CDK2, CDK4, and CDK6-mediated retinoblastoma protein (pRb) phosphorylation (He et al., 2007). p16 similarly prevents pRb inactivation, but in a p53-independent manner (Chen et al., 2006). pRb naturally binds E2F/DP transcription factor complexes to block transcription of E2F target genes, thus failure to phosphorylate pRb halts cell cycle progression from the G1 to S phase (Narita et al., 2003).

It is important to note that, while simplified here, the role for p53 in cell survival is complex and somewhat contradictory, as p53 activation can actually suppress senescence, instead causing cell quiescence (Demidenko et al., 2010) or apoptosis (reviewed in Salminen et al., 2011). In this regard, a cell’s fate might be decided by the amount of damage sustained, and the expression of other senescence-linked factors. Molecular understanding of senescence is complicated further by the fact that the relative contribution of p21, p16, and other cell cycle regulators is thought to be context dependent (van Deursen, 2014).

## Senescent Cell Characteristics

Morphologically, senescent cells exhibit flattened, elongated features, and may have multiple nuclei and enlarged vacuoles (Rhinn et al., 2019). Senescence-associated beta galactosidase is often used as an archetypal senescence biomarker (Dimri et al., 1995; Debacq-Chainiaux et al., 2009), yet its specificity has come under criticism (Krishna et al., 1999; Lee et al., 2006). For that reason, it is most often used in conjunction with other key biomarkers, such as p16 and p21, to confirm senescence (Baker et al., 2016; Matjusaitis et al., 2016; Biran et al., 2017).

Senescent cells may also possess regions of highly condensed chromatin (senescence-associated heterochromatic foci; Zhang et al., 2007) and DNA damage-induced chromatin alterations, including γH2AX and H3K9Me3 (Rodier and Campisi, 2011). Loss of histones, centrosome aberrations and the breakdown of the nuclear envelope (e.g., degradation of lamin B1) similarly occur in many senescent states to enable rearrangement of heterochromatin (Tigges et al., 2014; Wang et al., 2017). These chromatin modifications sequester E2F target genes to potentiate senescence (Shah et al., 2013). Moreover, senescence is reinforced by microRNA-mediated silencing of E2F target genes (Benhamed et al., 2012).

Experimental manipulation of epigenetic marks has demonstrably shown their importance in controlling the molecular induction of cellular senescence. H3K27me3, for example, represses p16 and p14 expression by silencing the INK4a-ARF locus (Kotake et al., 2007). Removal of H3K27me3, by JMJD3-induced demethylation (Agger et al., 2009; Sui et al., 2019) or pharmacological inhibition of the histone lysine methyltransferase, EZH2 (Ito et al., 2018), promotes p16 expression and senescence. Inhibition of EZH2 also leads to SASP production via enrichment of H3K27ac, and loss of H3K27me3, at SASP-related loci (Ito et al., 2018). Overexpression of another histone demethylase, UTX, can also silence H3K27me3 to promote cellular senescence (Perrigue et al., 2020).

Stressed cells are repressed at the transcriptional level to prevent the expansion of potentially harmful mutations. It is therefore understandable that regulators, such as p53, are not only responsible for initiating senescence, but also decide whether cells should instead enter temporary quiescence or undergo apoptosis (Salminen et al., 2011). Intriguingly, senescent cells may actually retain heightened resistance to apoptosis, first demonstrated in fibroblasts (Wang, 1995), possibly due to altered p53 signalling (Childs et al., 2014) and upregulation of pro-survival pathways (e.g., BCL-2 and ephrins, Zhu et al., 2015). Indeed, senescent keratinocytes are resistant to ultraviolet radiation-induced apoptosis (Chaturvedi et al., 2004) and senescent fibroblasts to thapsigargin-induced apoptosis (Ryu et al., 2007). Senescent endothelial cells, on the other hand, are more likely to undergo apoptosis than their non-senescent counterparts (Hampel et al., 2004). Clearly, this senescence trait is situational, and not ubiquitous to all cell types.

The hypersecretory phenotype of senescent cells is most often referred to as the senescence-associated secretory phenotype (SASP), an attribute closely linked to the positive or negative outcomes of tissue senescence that appears to be cell-type and context-dependent. Even though studies have characterised the SASP in multiple cell types, its detailed composition remains elusive. Broadly, the SASP comprises a collection of pro-inflammatory cytokines and chemokines, growth factors, proteases, lipids and extracellular matrix components (Freund et al., 2010; Elzi et al., 2012; Acosta et al., 2013; Lopes-Paciencia et al., 2019). It is thought to mainly be a feature of senescent cells that have undergone a DNA damage response, as a SASP is not apparent in cells that naturally senesce due to overexpression of p16 and p21 (Coppé et al., 2011). However, a DNA damage-independent SASP can occur in fibroblasts via p38MAPK phosphorylation, challenging previous preconceptions (Freund et al., 2011). Collectively, the secretome is the characteristic of senescent cells that confers most of their biological effects, significantly contributing to age-related functional decline (Rodier et al., 2009) and chronic disease (Zhu et al., 2014) in autocrine and paracrine manners.

The SASP is dynamically regulated by a number of factors that mostly converge on the NF-κB complex (Sun et al., 2018). Inflammatory cytokines, such as IL-1α, can form positive feedback loops with NF-κB and partner cascades to reinforce SASP release and senescence (Kuilman et al., 2008; Orjalo et al., 2009). In fact, multiple authors have demonstrated activation of NF-κB gene sets following senescence (Kuilman et al., 2010; Lujambio et al., 2013), while p53 and NF-κB are linked in coregulatory (in macrophages, Lowe et al., 2014) and antagonistic (HeLa cells, Huang et al., 2007) manners. The importance of NF-κB in senescence is highlighted by studies where NF-κB suppression allows oncogene-induced IMR-90 fibroblasts to bypass senescence (Chien et al., 2011) and reduces senescence in osteoarthritic cartilage (Wu et al., 2015).

**FIGURE 1:**
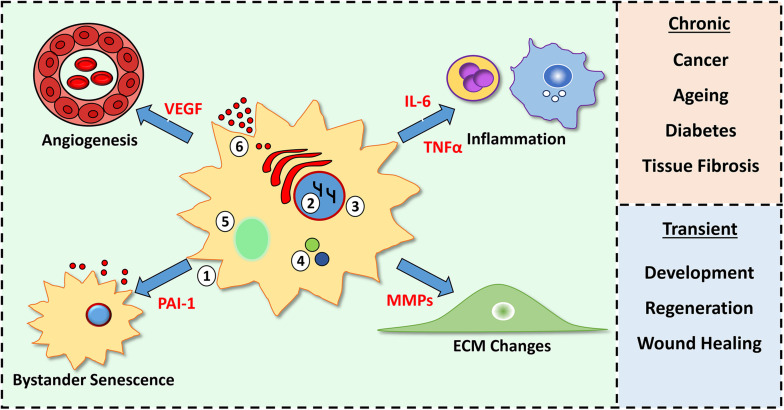
Characteristics of senescent cells and their tissue consequences. Senescent cells feature morphological changes **(1)**, chromatin modifications **(2)**, loss of nuclear envelope lamin B1 **(3)**, increased p16 and p21 **(4)**, senescence-associated beta galactosidase activity **(5)** and production of a senescence-associated secretory phenotype (SASP, **6**). SASP components (red) alter cellular processes (e.g., inflammation and angiogenesis) within the microenvironment. A chronic SASP causes negative outcomes, while a short-lived, transient SASP is beneficial.

Indeed, the SASP (e.g., TGFβ) can potentiate senescence in neighbouring cells (Acosta et al., 2013), but also promote senescent cell clearance by attracting immune cells (Kang et al., 2011; Tasdemir et al., 2016). The induction of senescence in, and clearance of, premalignant cells consequently reinforces tumour suppression. Paradoxically, the SASP can also drive pre-cancerous development in proximal tissues as many SASP proteins are potent mitogenic factors (e.g., VEGF, Coppé et al., 2006, 2010; Collado et al., 2007). The plasticity of the SASP across different microenvironments, cell types and stimuli (Campisi, 2013; Lupa et al., 2015; Maciel-Baron et al., 2016; Sun et al., 2018) further complicates our understanding of its role within tissues. However, it is clear that senescence, and the SASP, remain important regulators of normal physiology and pathology. Tissue consequences of senescent cells and their SASP are summarised in Figure 1.

## Roles for Senescence During Development and Regeneration

Many crucial biological processes require cells to undergo cell cycle arrest and differentiation to terminal states. For example, developmental lineage-specification requires cells to differentiate in a temporospatial manner in order to properly form tissues (Da Silva-Álvarez et al., 2019). In the skin, basal keratinocytes first proliferate, and then differentiate, transiting through the epidermis to replenish the non-viable stratum corneum (Fuchs and Byrne, 1994). In fact, to allow effective keratinocyte differentiation, p21 is activated initially (Missero et al., 1996), but then suppressed (Di Cunto et al., 1998). It is therefore unsurprising that tumour suppressor genes also aid development and regeneration through control of quiescence, terminal differentiation, apoptosis and senescence (e.g., Di Giovanni et al., 2006; Watkins et al., 2013).

Three main roles have been put forward for the presence of senescent cells during embryogenesis: (1) to promote the regression of transient structures; (2) to balance cell populations and/or; (3) to act as a signalling hub to regulate tissue morphogenesis (Da Silva-Álvarez et al., 2019). In embryonic development, temporal induction of senescence is required for tissue patterning in the developing limb bud. Here, p21 induction leads to SASP factor expression (e.g., FGF), stimulating cell proliferation and tissue formation. Resulting senescent (and apoptotic) cells are then effectively cleared by macrophages, prior to tissue remodelling. Indeed, genetic knockdown of p21 to attenuate senescence leads to mild patterning defects in murine limbs (Storer et al., 2013). In a corroborating study, p21 was shown to contribute to senescence-linked development in a p53-independent manner in human and murine embryos (Muñoz-Espín et al., 2013). In this case, however, loss of p21 was compensated for by increased apoptosis. Thus, p21-regulated senescence and apoptosis can perform synergistic roles during organismal development.

Akin to development, lower organisms and anamniotes are able to regenerate their tissues to full form and function, either as juveniles or throughout their lives (Brockes and Kumar, 2005). In fact, it has recently been shown that senescence may play an important role in these regenerative processes. In salamanders, senescence induction occurs at the intermediate stages of limb regeneration and then diminishes due to effective clearance by macrophages (Yun et al., 2015). Senescence is similarly invoked during pectoral fin regeneration in zebrafish, and impaired when senescence is blocked with the senolytic compound, ABT-263 (Da Silva-Álvarez et al., 2020). Given the importance of senescence in regulating tissue formation throughout development and regeneration, it is logical to ask whether senescence could play a role in the reparative responses of higher vertebrates.

## Senescence in Normal Tissue Repair

Tissue repair is necessary for all life. While it seldom leads to full regeneration, the process prevents exsanguination and infection, and aids structural and functional restoration required for survival. Tissue repair is rapid and highly dynamic, comprising multiple cell types and overlapping processes that broadly include haemastasis, inflammation, cell proliferation and dermal remodelling (Wilkinson and Hardman, 2017). During haemastasis, an insoluble blood clot is formed and endothelial cells from damaged vasculature enter the wound, depositing a temporary fibrin scaffold and releasing factors to attract both circulating immune cells and resident skin cells (Velnar et al., 2009). Inflammatory cells are rapidly recruited to the site of damage, first dominated by neutrophils and pro-inflammatory macrophages to remove bacteria and necrotic tissue (Young and McNaught, 2011). Later stage healing is characterised by a switch to anti-inflammatory macrophages, which phagocytose any remaining pro-inflammatory cells, supporting fibroplasia and wound resolution (Korns et al., 2011). To allow effective repair, keratinocytes undergo partial epithelial-to-mesenchymal transition and begin migrating to close the wound gap, a process known as re-epithelialisation (Shaw and Martin, 2016). Formation of new vasculature (angiogenesis) is essential to provide sustenance during the highly proliferative stage of healing (Baum and Arpey, 2005). Finally, the immature matrix laid down during early healing is replaced by stronger scaffold proteins, such as mature collagen fibres produced and remodelled by fibroblasts (Li et al., 2007). Each stage of wound repair involves extensive cellular communication, orchestrated by cytokines, chemokines, growth factors and components of the extracellular milieu. The plasticity of the response, and the cellular behaviours that occur, are homologous to those observed in cancer (e.g., immune cell infiltration, invasion and epithelial-to-mesenchymal transition, Schäfer and Werner, 2008). It is therefore not unreasonable to suggest that senescence, and associated mechanisms, could significantly contribute to wound healing.

Indeed, pertinent roles for senescence in tissue injury have been emerging, largely focusing on the beneficial, transient initiation of senescence during repair. Here, induction of senescence following liver damage (Krizhanovsky et al., 2008) and cutaneous injury (Jun and Lau, 2010) was shown to prevent excessive fibrosis that would otherwise cause tissue dysfunction. Krizhanovsky et al. (2008) confirmed that reduced fibrosis was the result of senescence-linked fibrolytic enzyme production, and immune-regulated clearance of injury-expanded cell populations that would otherwise contribute to excessive matrix deposition. Likewise, senescence decreased fibrosis in a model of cardiac injury, where genetic ablation of p53 and p16 accelerated fibrosis (Meyer et al., 2016). Ectopic expression of Ccn1, which increased cardiac senescence, also limited fibrosis in this model. Interestingly, Jun and Lau (2010), the first authors to observe transient senescence during skin repair, revealed that Ccn1 causes fibroblast senescence via an oxidative-stress dependent mechanism. Upregulation of Ccn1 was vitally important to prevent excessive fibrosis. More recently, the same authors demonstrated that topical application of another Ccn family member, Ccn2, similarly actuates senescence and reduces fibrosis in cutaneous murine wounds (Jun and Lau, 2017).

By contrast, when Demaria et al. (2014) ablated p16- and p21-expressing cells in mice they observed impaired extracellular matrix deposition and a decreased rate of wound closure. Intriguingly, by day 15 post-injury, these senescent-deficient wounds were excessively fibrotic. Similar to previous research (Jun and Lau, 2010), transient senescence appeared limited to fibroblast-like cells, which produced a PDGFA-enriched SASP to stimulate appropriate skin repair (Demaria et al., 2014). Studies continue to explore the importance of transient senescence during acute wound healing, with Hiebert et al. (2018) recently reporting that overexpression of nrf2 promotes fibroblast senescence, which is accompanied by accelerated wound re-epithelialisation and extracellular matrix deposition. Although at present limited to murine models, these key investigations provide clear evidence that temporal induction of senescence is necessary for effective skin repair. Yet, many questions remain unanswered. For instance, does transient wound-induced senescence arise through intrinsic cell factors or environmental influences? And how are these senescent cells so effectively cleared once they are no longer required?

## Senescence in Aged and Diabetic Wound Healing

The above studies provide substantial insight into the importance of senescence for the healing of experimental wounds. What they do not address is the potential differential influences of acute vs. chronic senescence to tissue repair, nor how senescence could be involved in pathological healing. These are important considerations for the clinical setting, where effective healing can mean the difference between life or death (Han and Ceilley, 2017). Chronic, non-healing wounds are a huge socioeconomic burden, reducing quality of life and costing billions each year to treat (Guest et al., 2015). Considered a “silent epidemic” (Lindholm and Searle, 2016), chronic wounds display diverse aetiology, with incomplete molecular and cellular understanding (Frykberg and Banks, 2015). Inadequate current treatments mean it is fundamentally important to further understand why chronic wounds fail to heal, and ultimately develop more effective therapies.

It has long been appreciated that chronic wound pathology is almost entirely restricted to those who are elderly and/or diabetic. This is fascinating, as the biological processes of ageing and diabetes are themselves notably linked to senescence (Wilkinson and Hardman, 2020). Senescence is both a characteristic feature of Baker et al. (2013), Xu et al. (2015), and Helman et al. (2016) and contributor to Baker et al. (2008, 2011) widespread tissue ageing. Epigenetic modifications are one feature of ageing that is linked to senescence. Genomic instability and DNA methylation changes correlate with chronological ageing in mice (Stubbs et al., 2017) and humans (Horvath, 2013). Interestingly, the repressive mark, H3K27me3, showed altered DNA coverage on aged vs. young stem cells (Liu et al., 2013; Sun et al., 2014), which may contribute to their reduced renewal capacity.

Another attribute of normal metabolic ageing that is experimentally linked to senescence is cumulative oxidative damage. For example, human diploid fibroblasts (Duan et al., 2005) and endothelial cells (Ruan et al., 2014) undergo senescence in the presence of heightened reactive oxygen species (ROS), while replicative lifespan can be extended in cell culture by lowering oxygen tension (Parrinello et al., 2003). More notably, exposure to ultraviolet radiation simulates photoageing by increasing ROS production in skin (Herrling et al., 2006), while ROS upregulates p16 in skin cells (Jenkins et al., 2011). Skin ageing is also characterised by cell accumulation of p16 (Waaijer et al., 2012) and senescence-associated beta galactosidase (Dimri et al., 1995; Ressler et al., 2006). This association is causally corroborated by Xu et al. (2018), who demonstrated that transplantation of senescent cells to young mice accelerated ageing, while Baker et al. (2011) revealed that eradication of p16-positive cells alleviated features of premature ageing in a murine progeroid model.

The link between diabetes and senescence is less well-established, but is an area of intense current research. As previously mentioned, senescent cells cause widespread disruption to normal tissue architecture by virtue of their SASP (Coppé et al., 2010). Major SASP constituents influence senescence by targeting immunological pathways, such as NF-κB (Salminen et al., 2011). This leads to matrix proteolysis and increased inflammation, primary features of aged and diabetic wounds (Makrantonaki et al., 2017; Wilkinson et al., 2019c). Indeed, growing evidence suggests that a heightened intrinsic immune response, or “sterile” inflammation, contributes to age- and diabetes-related pathology (reviewed in Prattichizzo et al., 2016). Characteristic features of diabetes that drive immune cell accumulation, and therefore potentiate senescence, include obesity and hyperglycaemia (Yokoi et al., 2006; Minamino et al., 2009; Maeda et al., 2015; Schafer et al., 2016). These processes most likely promote senescence via increasing advanced glycation end-products and causing widespread oxidative damage (Coughlan et al., 2011; Fang et al., 2016).

Turning specifically to the skin, it is clear that in diabetic and aged tissue, accumulation of senescent cells extends to both uninjured skin and wounds (Ressler et al., 2006; Waaijer et al., 2012; Wilkinson et al., 2019a). Previous authors have demonstrated that chronic venous leg ulcers harbour senescent fibroblasts (Mendez et al., 1998; Vande Berg et al., 1998; Agren et al., 1999; Wall et al., 2008). The presence of senescent fibroblasts in chronic wounds may even exacerbate pathology, where it was shown that ulcers containing over 15% senescent cells were hard to heal (Stanley and Osler, 2001). We recently reported a novel mechanistic link between senescence and healing in diabetic wounds (Wilkinson et al., 2019a). Here, intrinsically senescent macrophages were observed to promote impaired wound healing in a non-aged, murine model of diabetic pathological repair.

Indeed, many SASP factors attract monocytes and macrophages (e.g., MCP-1; Kamei et al., 2006; Coppé et al., 2008; Prattichizzo et al., 2018), often with a pro-inflammatory phenotype (Mosser and Edwards, 2008; Lujambio et al., 2013). Excessive immune cell recruitment and inappropriate retention is a hallmark of chronic wound pathology. This may be even be exacerbated by other local factors, such as iron, which induces a pro-inflammatory phenotype in macrophages and leads to fibroblast senescence in chronic venous leg ulcers (Sindrilaru et al., 2011). Thus, macrophages are likely a nexus for uncontrolled local inflammation in both diabetic pathogenesis and senescence, ultimately delivering poor wound healing. Moreover, the impaired function of macrophages (and other immune cell types) in aged (Swift et al., 2001) and diabetic (Wilkinson et al., 2019b) wounds likely contributes to prolonged, rather than transient, senescence due to ineffective clearance mechanisms.

Senescence in the wound environment is probably not limited to fibroblasts and macrophages, as other wound cells, including keratinocytes (Smirnov et al., 2016) and endothelial cells (Ruan et al., 2014), are capable of undergoing senescence in response to environment cues. Senescent keratinocytes are certainly observed in aged skin (Velarde et al., 2012) and are suggested to influence the reduced regenerative capacity of aged epidermis (Zouboulis et al., 2008). Chronic wounds also harbour pathogenic microorganisms (Kalan et al., 2019) that may contribute to senescence by stimulating ROS production in keratinocytes and exacerbating inflammation (Grange et al., 2009). Indeed, this may occur via specific bacterial virulence factors, as pyocyanin from *Pseudomonas aeruginosa* can induce senescence in fibroblasts (Muller et al., 2009).

It is clear that the chronic ulcer milieu, which is rich in pro-inflammatory factors, indirectly causes senescence via exacerbating inflammation. However, as wound fluid from venous leg ulcers directly induces senescence in neonatal fibroblasts (Mendez et al., 1999), it is likely that the local microenvironment also stimulates cellular senescence. Intrinsically senescent wounds cells, such as fibroblasts, are similarly capable of potentiating senescence across neighbouring cell types in a paracrine manner, via their SASP (Acosta et al., 2013; Wilkinson et al., 2019a). Moreover, in local environments where the SASP is insufficient to directly induce cellular senescence, it may still promote pathological cellular phenotypes, such as epidermal hyperproliferation (Albanesi et al., 2018) and excessive dermal proteolysis (via MMPs; Caley et al., 2015).

**FIGURE 2:**
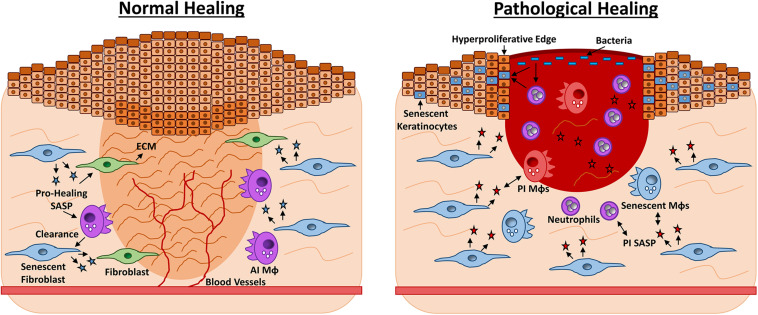
Roles for senescence in acute vs. chronic wound repair. Late-stage wound healing is characterised by extracellular matrix (ECM) deposition, full wound closure and blood vessel perfusion **(left)**. Senescent fibroblasts appear during late-stage healing and contribute to ECM deposition by producing a pro-healing senescence-associated secretory phenotype (SASP, blue stars) containing PDGFA. Senescent fibroblasts prevent excessive fibrosis via CCN1 and CCN2. The senescent cells are then effectively cleared by anti-inflammatory macrophages (AI MΦs) and the skin is restored. Chronic healing wounds feature wound edge hyperproliferation and excessive inflammation **(right)**. Here senescent fibroblasts and MΦs (blue) exacerbate inflammation via a pro-inflammatory (PI) SASP of cytokines and proteases. The SASP may also exert a paracrine effect, causing senescence in other wound cell types. High bacterial load stimulates further inflammation and oxidative stress, which can cause hyperproliferation or senescence in keratinocytes. Senescent cells are not effectively cleared by the dysfunctional chronic wound immune cells, thus tissue damage persists and the wound fails to heal.

To add a further level of complexity, evidence for the disparities between transient and chronic senescence is beginning to emerge, with clear implications for wound healing. For instance, stemness and reprogramming in keratinocytes is promoted by a transient SASP, yet inhibited when the SASP becomes chronic (Ritschka et al., 2017). Transient senescence also encourages matrix deposition following tissue injury (Demaria et al., 2014), but prevents excessive fibrosis (Jun and Lau, 2010), while chronic senescence is linked fibrotic disease (Yanai et al., 2015). Taken together, published and emerging studies are certainly challenging the dogma that senescence is primarily limited to age-related dysfunction and cancer. Indeed, evolving understanding of the concept of transient vs. chronic senescence is likely to deliver important new insight into the processes that occur during acute and pathological repair. Current understanding of senescence contribution to normal and pathological wound healing is summarised in Figure 2.

## Cellular Senescence as a Therapeutic Target in Pathological Wounds

The widespread causative biological effects of cellular senescence in tissue ageing pathology make the therapeutic modulation of senescence an attractive target for a plethora of age-related diseases. Genetic studies positively support this idea, with inducible knockdown of p16 alleviating hallmark features of ageing in progeroid murine models (Baker et al., 2011; Sato et al., 2015). In fact, the well-documented effects of caloric restriction, which both extends mammalian lifespan (Sohal and Weindruch, 1996) and delays the onset of age-related disease (Weindruch and Walford, 1982; Colman et al., 2014), may be a physical manifestation of tissue senescence modulation. Caloric restriction has been shown to reduce cardiac senescence (Shinmura et al., 2011), and senescence in hepatocytes and intestinal crypt cells *in vivo* (Wang et al., 2010). At the epigenetic level, caloric restriction protects against age-related changes in DNA methylation (Hahn et al., 2017). Caloric restriction also decreases senescence partly by upregulating the epigenetically linked sirtuin pathway, promoting anti-apoptosis and anti-inflammatory mechanisms (Bonda et al., 2011). Subsequent effects include slowing metabolic processes that contribute to cellular ageing (e.g., oxidative stress, Yang et al., 2016), increasing antioxidant production (Meydani et al., 2011), and increasing autophagy to remove damaged and unimportant intracellular components (reviewed in Cuervo, 2008). Moreover, sirtuins may play important roles in preventing age-related decline in skin repair, as SIRT1 deficiency exacerbates healing pathology in diabetic wounds (Thandavarayan et al., 2015).

Although caloric restriction (without malnutrition) provides a multitude of health benefits, it retains poor feasibility as a clinical intervention, requiring high compliance and patient discipline. Many lifestyle choices, such as obesity, are actually strongly associated with social status (Drewnowski and Specter, 2004). Similarly, those suffering from uncontrolled type II diabetes and severe chronic wounds are often from socially deprived backgrounds (Anderson et al., 2018), a difficult population in which to manage compliance. For all of these reasons, a considerably more attractive proposition is the use of senescence-targeted drugs, otherwise known as senolytics. These drugs affect unique features of senescent cells, such as resistance to apoptosis (Salminen et al., 2011). Senescent cells upregulate prosurvival pathways, particularly BCL-2 (Ovadya and Krizhanovsky, 2018). This opens up drug repurposing opportunities around the numerous BCL-2 inhibitors that were developed for the treatment of cancer (Roberts et al., 2016; Montero and Letai, 2018). Results have been promising. Targeting BCL-2 *in vivo* induces apoptosis and thus eliminates senescent cells in the lung following irradiation (Yosef et al., 2016) and throughout the body following irradiation or natural ageing (Chang et al., 2016). Chang et al. (2016) further established that senescent human and murine fibroblasts, and human renal epithelial cells, are more susceptible to BCL-2 inhibitor (ABT-263) than non-senescent cells, proposing potent and specific effects. Unfortunately, traditional BCL-2 inhibitors possess activity against other BCL class proteins, such as BCL-XL, raising questions around off-target effects in the clinic, including thrombocytopenia and neutropenia. As a result, more specific BCL-2 inhibitors with lower toxicity are being tested (King et al., 2017). It has even been suggested that low-dose, combinatorial use of senolytics may be an effective and less harmful alternative (Ovadya and Krizhanovsky, 2018).

Other senolytics that have demonstrated experimental efficacy include the tyrosine kinase inhibitor, Dasatinib, used to treat leukaemia (Keskin et al., 2016), and the flavonoid p53 activator, Quercetin (Khan et al., 2016). Combinatorial treatment with Dasatinib and Quercetin extends lifespan, alleviates frailty (Xu et al., 2018), and improves vasomotor function (Roos et al., 2016) in aged mice. Dasatinib and Quercetin have also shown promise in a phase I trial in diabetic kidney disease patients, where reduced senescent cells and circulating SASP factors were observed following administration (Hickson et al., 2019). Alternative flavonoids are now being tested for their potential senolytic effects, such as Fisetin, which is able to eliminate senescent cells and, crucially, restore tissue function in aged mice (Yousefzadeh et al., 2018).

The importance of transient senescence for effective healing should not be underestimated. As noted previously, temporary induction of senescence aids rapid tissue reformation (Demaria et al., 2014; Hiebert et al., 2018). During a normal damage response, these senescent cells are effectively cleared by natural killer cells (Krizhanovsky et al., 2008) and macrophages (Yun et al., 2015). Nevertheless, in chronic situations, senescent cells persist, likely due to elevated immunosenescence and resulting impaired immunological functions (Hall et al., 2016). It follows that treatments to boost immune system function, for instance by aiding senescent cell recognition, could be beneficial in the context of transient senescence and tissue repair. Generally, senescent cells express stimulatory ligands that bind to NK2GD receptors on natural killer cells, thus initiating a killing response (Sagiv et al., 2016). However, senescent fibroblasts in aged skin have recently been shown to express HLA-E, which bypasses recognition and clearance by natural killer and T cells (Pereira et al., 2019). Here, approaches developed in the cancer field may also be useful, for example engineering T cells to express receptors that target specific cellular (tumour) proteins (reviewed in June et al., 2018). Studies to identify and validate new senescent cell receptors will be essential to the development and clinical application of such immune-regulated approaches.

Indeed, the emergence of global profiling methodologies, such as single-cell RNA sequencing, could provide the basis to understanding senescence-linked changes in ageing and pathology by identifying unique cell-based transcriptomic signatures within tissues. Kimmel et al. (2019) used this approach to compare cell frequency, heterogeneity and age-related transcriptomic changes between aged and young murine tissues. Similarly, Angelidis et al. (2019) combined transcriptomics and proteomics to not only identify the epigenetic and transcriptional consequences of ageing in the lung, but also determine their functional implications. Future harnessing of these technologies could therefore facilitate the identification and targeting of key senescence-linked receptors and biomarkers in a tissue and pathology-specific manner.

**FIGURE 3:**
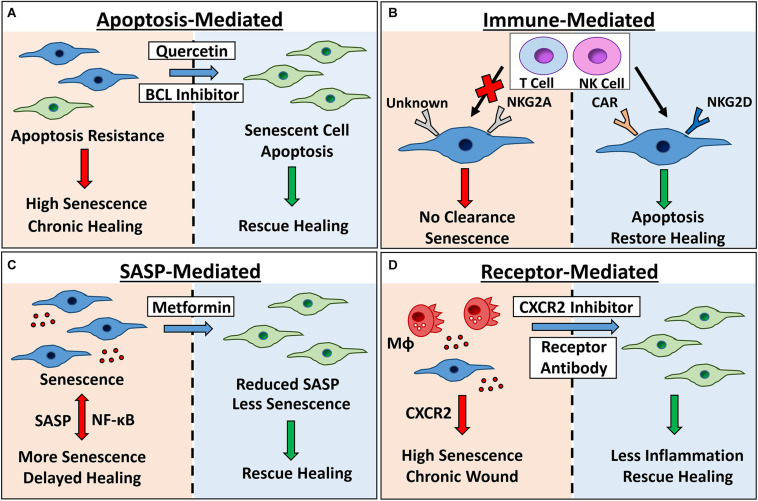
Therapeutic targeting of senescence for chronic healing wounds. Senescent cells accumulate in chronic healing wounds, contributing to inflammation and poor healing. Senescence can be targeted by: **(A)** inhibiting pro-survival pathways with BCL inhibitors and broad spectrum drugs (e.g., quercetin) to cause apoptosis; **(B)** engineering chimeric antigen receptor (CAR) T cells to target senescent cell receptors, or modulating expression of natural killer (NK) cell receptors NKG2A and NKG2D to increase clearance; **(C)** using Metformin or other SASP inhibitors to reduce NF-κB-mediated inflammation and bystander senescence and; **(D)** inhibiting receptors known to potentiate wound senescence (e.g., CXCR2). Red arrows/left panels = negative outcomes. Green arrows/right panels = positive outcomes. MΦ= macrophage. Senescent cells = blue.

Alternative strategies to diminish or limit senescence and alleviate pathology instead target the SASP or specific senescence-linked receptors directly (summarised in Figure 3). Certainly, the SASP is transcriptionally regulated by NF-κB and others (Salminen et al., 2011), and contributes heavily to tissue deterioration, both driving widespread destruction and reinforcing senescence (Rodier et al., 2009; Acosta et al., 2013). SASP inhibitors affect key transcriptional mediators, blocking signalling and preventing SASP production (Moiseeva et al., 2013). Interestingly, Metformin, a widely used anti-diabetic drug, is an effective SASP inhibitor (reviewed in Rena et al., 2017) able to directly accelerate healing in diabetic mice (Han et al., 2017). Rapamycin, another SASP inhibitor, was the first drug revealed to extend lifespan in mice (Harrison et al., 2009), and enhance the replicative lifespan of human keratinocytes (Horvath et al., 2019) and skin fibroblasts *in vitro* (Sodagam et al., 2017). Although these studies suggest potential skin-related benefits of SASP inhibitors, removal of the SASP could be deleterious, impairing the healing response and preventing senescent cell clearance (von Kobbe, 2019). Consequently, it may be more advantageous to target particular SASP components known to impact tissue function, either with antibodies (e.g., IL-1α, Orjalo et al., 2009), or specific inhibitors (e.g., against CXCR2, Wilkinson et al., 2019a).

We remain a long way from implementing senescence-targeted treatments for pathological wound healing, yet it is reassuring to see that current senolytic drugs display efficacy across a wide range of tissues and pathologies. In a number of studies, systemic senolytic treatments have been shown to have clear effects in peripheral target tissues across a range of treatment regimens. For example, a single dose of BCL inhibitor (Yosef et al., 2016), and dosing over consecutive days (Chang et al., 2016), was able to reverse irradiation-induced senescence in different tissues. In the work by Xu et al. (2018), aged mice showed improved physical performance following biweekly oral treatments of Dasatinib and Quercetin for 4 months, yet reduced SASP was observed in human *ex vivo* cultured adipose tissue within 48 h of treatment. Moreover, a single 3 day oral treatment of Dasatinib and Quercetin was able to reduce senescence in the adipose tissue of diabetic patients in a phase I trial (Hickson et al., 2019). These studies therefore suggest that senolytic treatments not only have rapid effects in target peripheral tissues, but can overcome established tissue senescence.

Experimental studies do show beneficial effects of modulating senescence in the skin. For example, elimination of senescent cells from the epidermis restored proliferative capacity in hair follicle stem cells (Yosef et al., 2016), known to participate in wound healing (Joost et al., 2018). Further, blockade of the potential senescence receptor, CXCR2 (Acosta et al., 2008), directly accelerated healing in human *ex vivo* skin wounds and diabetic murine wounds *in vivo* (Wilkinson et al., 2019a). Here, a CXCR2 antagonist was administered to wounds topically (*ex vivo*) and subcutaneously (*in vivo)*, suggesting direct delivery to the wound site as a viable administration route. Indeed, elevated CXCR2 has previously been observed in diabetic wounds (Wetzler et al., 2000), and more recently in T cells from human diabetic patients (Lau et al., 2019). We note with interest that pharmacological inhibition of CXCR1/2 additionally prevents inflammation-mediated damage to pancreatic islets, thus prohibiting streptozocin-induced diabetes in mice (Citro et al., 2015). Therefore, CXCR2 appears a common factor in both the ontology and local pathology of diabetes. Senolytics should certainly be considered for the treatment of human chronic wounds characterised by high levels of senescence (Stanley and Osler, 2001). However, given that knockdown of CXCR2 (Devalaraja et al., 2000) and ablation of senescent cells (Demaria et al., 2014) actually delays acute wound healing, future senescence-targeted therapies should be reserved for the treatment of chronic conditions.

## Conclusion

Despite seemingly contradictory roles in many cancers, the detrimental contribution of cumulative senescence to ageing and age-related disease is now well-established. By contrast, the short-lived, transient senescence observed to benefit tissue development, regeneration and repair, remains significantly less well-characterised. In wound repair, a paradigm is emerging where local transient senescence predominately constrains fibrosis, while chronic senescence drives diabetic wound pathology. Indeed, experimentally blocking the senescence-linked receptor, CXCR2, *in vivo* reverses pathology and accelerates diabetic healing. These observations now pave the way to explore the beneficial effects of senescence-targeted therapies for the treatment of chronic wounds.

## Author Contributions

HW wrote the manuscript and prepared the figures. MH provided critical appraisal. Both authors contributed to the article and approved the submitted version.

## Conflict of Interest

The authors declare that the research was conducted in the absence of any commercial or financial relationships that could be construed as a potential conflict of interest.
